# Physiological versatility of ANME-1 and Bathyarchaeotoa-8 archaea evidenced by inverse stable isotope labeling

**DOI:** 10.1186/s40168-024-01779-z

**Published:** 2024-04-03

**Authors:** Xiuran Yin, Guowei Zhou, Mingwei Cai, Tim Richter-Heitmann, Qing-Zeng Zhu, Mara Maeke, Ajinkya C. Kulkarni, Rolf Nimzyk, Marcus Elvert, Michael W. Friedrich

**Affiliations:** 1 State Key Laboratory of Marine Resource Utilization in South China Sea, Hainan University, Renmin Ave. No.58, Haikou, 570228 China; 2 Microbial Ecophysiology Group, Faculty of Biology/Chemistry, University of Bremen, James-Watt-Strasse 1, Bremen, D-28359 Germany; 3 Max Planck Institute for Marine Microbiology, Bremen, Germany; 4 MARUM-Center for Marine Environmental Sciences, University of Bremen, Leobener Straße 8, Bremen, D-28359 Germany; 5 School of Resources and Environmental Engineering, Anhui University, Hefei, Anhui China; 6 Institute of Chemical Biology, Shenzhen Bay Laboratory, Shenzhen, China; 7 Archaeal Biology Center, Institute for Advanced Study, Shenzhen University, Shenzhen, China; 8 Faculty of Geosciences, University of Bremen, Bremen, Germany

**Keywords:** Trophic strategy, Inverse labeling, Stable isotope probing, ANME-1, Bathy-8

## Abstract

**Background:**

The trophic strategy is one key principle to categorize microbial lifestyles, by broadly classifying microorganisms based on the combination of their preferred carbon sources, electron sources, and electron sinks. Recently, a novel trophic strategy, i.e., chemoorganoautotrophy—the utilization of organic carbon as energy source but inorganic carbon as sole carbon source—has been specifically proposed for anaerobic methane oxidizing archaea (ANME-1) and Bathyarchaeota subgroup 8 (Bathy-8).

**Results:**

To further explore chemoorganoautotrophy, we employed stable isotope probing (SIP) of nucleic acids (rRNA or DNA) using unlabeled organic carbon and ^13^C-labeled dissolved inorganic carbon (DIC), i.e., inverse stable isotope labeling, in combination with metagenomics. We found that ANME-1 archaea actively incorporated ^13^C-DIC into RNA in the presence of methane and lepidocrocite when sulfate was absent, but assimilated organic carbon when cellulose was added to incubations without methane additions. Bathy-8 archaea assimilated ^13^C-DIC when lignin was amended; however, their DNA was derived from both inorganic and organic carbon sources rather than from inorganic carbon alone. Based on SIP results and supported by metagenomics, carbon transfer between catabolic and anabolic branches of metabolism is possible in these archaeal groups, indicating their anabolic versatility.

**Conclusion:**

We provide evidence for the incorporation of the mixed organic and inorganic carbon by ANME-1 and Bathy-8 archaea in the environment.

Video Abstract

**Supplementary Information:**

The online version contains supplementary material available at 10.1186/s40168-024-01779-z.

## Background

Microbes are physiologically classified according to their modes of carbon, energy, and electron utilization, as in, e.g., chemoorganoheterotrophy or photoautotrophy. This key concept to define categories of similar lifestyles has been often used to characterize uncultivated microbes based on their metagenomically reconstructed genetic content [1–3]. The identification of trophic strategy is even more crucial for uncultivated archaea due to the severe knowledge gaps regarding their ecophysiology in the environment.

In recent years, a novel and unique nutritional category, chemoorganoautotrophy has been proposed for two archaeal groups: anaerobic methanotrophic archaea subgroup 1 (ANME-1) and Bathyarchaeota subgroup 8 (Bathy-8) [4, 5]. In these archaea, organic substrates such as methane and lignin are utilized solely as energy sources, while dissolved inorganic carbon (DIC) is assimilated autotrophically [4, 5]. Characterization of this trophic strategy is surprising because these archaea, as autotrophs, would have to have a disjointed pathway between energy metabolism (using organic carbon) and inorganic carbon assimilation, meaning that the basic amphibolic pathways [6, 7] are not present in these organisms. Nonetheless, the strategy of chemoorganoautotrophy has been demonstrated in biotechnologically engineered strains [8, 9]. In these synthetic autotrophic organisms, key genes connecting dissimilatory and assimilatory pathways are deleted, and CO_2_ fixation pathways are added to facilitate autotrophic capabilities. Although a few studies have classified some bacterial strains as chemoorganoautotrophs, their utilization of C1 organic substrates or methyl groups (formic acid, methanol, and choline) identifies them as C1-compound utilizers rather than chemoorganoautotrophs [10–12].

Without genetic engineering modifications of anabolic carbon utilization pathways, it seems unlikely for naturally occurring microbes to thrive as organoautotrophs. In general, microbes have amphibolic pathways that link energy metabolism and carbon assimilation, and thus, inorganic and organic carbon sources will be mixed within the cell [6, 7]. Furthermore, it is difficult to identify carbon utilization patterns accurately for as yet uncultivated microbes such as ANMEs and Bathyarchaeota, thus, requiring to test organoautotrophy of these microbes in environmental samples. Considering such low possibility of chemoorganoautotrophic activity in environments, we hypothesize that catabolic and anabolic pathways in ANME-1 and Bathy-8archaea are most likely linked. Thus, we propose that organic carbon could be transferred into cell carbon during energy metabolism, meaning that mixed carbon sources from both organic and inorganic carbon can contribute to the cell biomass rather than strict autotrophy. In order to address this hypothesis, we tracked organic carbon incorporation by nucleic acid-based stable isotope probing (SIP) in the presence of a high background of ^13^C-DIC. In combination with metagenomics, the evidences show that ANME-1 and Bathy-8 archaea may utilize both organic and inorganic carbon for their growth instead of purely relying on CO_2_ fixation.

## Methods

### Sediment incubation setup for SIP

Sediment retrieved from the Helgoland mud area (54° 05.23′ N, 007° 58.04′ E) by gravity coring during the RV HEINCKE cruise HE443 were used for SIP incubations in 2015. Sediment gravity cores were kept at 4 °C on board and then were cut into 25 cm sections. The fresh sediment was stored in 2.6-L jars at 4 °C with anoxic artificial sea water and headspace of N_2_. The geochemical profiles of the sediment were described in a previous study [13]. For SIP incubations conducted in 2017, sediment belonging to methanic zone from the depth of 238–263 cm was homogenized with artificial water (w:v = 1:4, 50 mL; 26.4 g L^−1^ NaCl, 11.2 g L^−1^ MgCl_2_·6H_2_O, 1.5 g L^−1^ CaCl_2_·2H_2_O, and 0.7 g L^−1^ KCl) and filled into sterile 120-mL serum bottles which were sealed with butyl rubber stopper. The slurry was vacuumed 3 times for 3 min in order to remove O_2_, and the headspace of culture was flushed with N_2_ at 1.5 atm as described previously [14]. Substrates added included lignin (30 mg/l; Sigma–Aldrich, USA—5% moisture), cellulose (30 mg/l; Sigma–Aldrich, USA—microcrystalline), and methane (50% in headspace; Linde, Germany—99.99%), lepidocrocite (30 mM; LanXess GmbH, Germany) with different combinations after a 10-day preincubation. All incubations were setup with triplicates. For inverse labeling, high concentration of 99% ^13^C-labeled bicarbonate (10 mM; Cambridge Isotope Laboratories, Tewksbury, Massachusetts, USA) was amended. This starting concentration achieved a ^13^C-labeling level of ~80% in DIC (dissolved inorganic carbon) in slurry based on the measurement CO_2_ concentrations in headspace (control, 45.35 ± 3.09 µM; ^13^C-DIC treatment: 213.46 ± 6.59 µM), which was still high at the end the incubations (cellulose + Lep. 67.32 ± 1.16%; lignin + Lep: 63.28 ± 4.57%) that can facilitate density shift to the heavy fractions for nucleic acid SIP [15]. This concentration of DIC (10 mM) will not substantially affect microbial community since DIC concentration from pore water of raw sediment at the same depth is ~7.5 mM [16]. The triplicate incubations were harvested after 255 and 386 days for RNA-SIP and DNA-SIP, respectively (see Table 1 for the reason of using RNA-SIP and DNA-SIP at different time points).

**Table 1:** The reason of using RNA-SIP and DNA-SIP for studying ANME-1 and Bathy-8, respectively

SIP	Sensitivity	Downstream metagenomics	MAG availability	Archaeal group
RNA-SIP	High	MAGs cannot be retrieved from RNA	Yes	ANME-1
DNA-SIP	Relatively low	Possible to retrieve MAGs from the DNA-SIP heavy fractions	No (Bathy-8 MAG was not retrieved from sediment)	Bathy-8

### Nucleic extraction, isopycnic centrifugation, gradient fractionation, and 16S rRNA gene sequencing

Nucleic acids were extracted according to Lueders et al. [17]. Briefly, 2 mL of slurry was transferred and centrifuged at 20,000 *g* for 5 min, and the wet sediment without supernatant was then collected from biological triplicates, which was used for cell lysis by bead beating, nucleic acid purification by phenol-chloroform-isoamyl alcohol extraction and precipitation with polyethylene glycol. For the RNA extract, DNA was removed by using the RQ1 DNase kit (Promega, Madison, WI, USA). DNA and RNA were quantified fluorimetrically using Quant-iT PicoGreen and Quant-iT RiboGreen (both Invitrogen, Eugene, OR, USA), respectively. In order to obtain enough nucleic acids for SIP, a volume of 30- to 50-ml slurry from each bottle was used for both RNA and DNA extraction separately.

Isopycnic centrifugation and gradient fractionation were performed to separate ^13^C-labeled from unlabeled nucleic acids as previously described [17]. Nucleic acid extracts from individual triplicate bottles were pooled to make up for low yields. We used RNA-SIP for the study of ANME-1 due to its high sensitivity while using DNA-SIP for Bathy-8 owing to the possibility of retrieving Bathy-8 MAGs from the heavy DNA-SIP fractions instead of the original sediment. Briefly, DNA and RNA were centrifuged at 192,600 *g* (48 h) and 124,000 *g* (65 h) after mixing with CsCl and CsTFA, respectively. After ultracentrifugation, 13 fractions were obtained. For RNA-SIP, cDNA from fractions 4 and 5 (heavy—1.810–1.820 g/ml), 6 and 7 (middle—1.800–1.809 g/ml), 8 and 9 (light—1.790–1.799 g/ml), as well as 10 and 11 (ultra-light—1.780–1.789 g/ml) were combined for sequencing. The heavy and light fractions were defined based on the nucleic acid amount quantified fluorimetrically in each fraction of the SIP runs with mixed ^12^C- and ^13^C nucleic acid (Fig. S1). In brief, the RNA-SIP profiles of the *E. coli* standard had the unlabeled peak at a density of ~1.790 g/ml and the fully ^13^C-labeled peak at ~1.823 g/ml; and the DNA-SIP profiles had the unlabeled DNA peaked at 1.696 g/ml and the fully labeled DNA peaked at 1.712 g/ml (Fig. S1). PCR was performed with a KAPA HiFi HotStart PCR kit (KAPA Biosystems, Cape Town, South Africa) and barcoded archaeal primer Arc519F (5′-CAGCMGCCGCGGTAA-3′) [18] and Arch806R (5′-GGACTACVSGGGTATCTAAT-3′) [19] as well as bacterial primer Bac515F (5′-GTGYCAGCMGCCGCGGTAA-3′) and Bac805R (5′-GACTACHVGGGTATCTAATCC-3′) [20]. Thermocycling was as follows: 95 °C for 3 min; 35 cycles at 98 °C for 20 s, 61 °C for 15 s, and 72 °C for 15 s; 72 °C for 1 min. PCR products were purified using the Monarch PCR Cleanup Kit according to the manufacturer. Equimolar amounts of PCR products per sample were combined. Amplicons were sequenced using the Illumina Hiseq 4000 platform with 150-bp paired-end reads at GATC Biotech (Konstanz, Germany). Raw reads were processed using the QIIME 1.9.0 software package [21]. Briefly, barcodes were extracted from the merged file of forward and reverse reads, followed by sequence demultiplexing to obtain a fastq file containing sequencing and sample information. Sequence reads with forward primer (Arch519F) were selectively sorted and then truncated to 143 bp, and low-quality reads with expected errors of more than 0.5 were discarded by quality filtering on USEARCH 10 [22]. After sequence dereplication and removal of singletons by USEARCH 10, OTUs were clustered at 97% identity using the UPARSE-OTU algorithm [23]. Taxonomy was assigned to OTUs based on the Silva database (Silva 132 [24]) on UCLUST [22] with 97% identity threshold. The OTU table and taxonomy assignment files were merged to obtain the final OTU table [25].

### Phylogenetic analyses

Archaeal 16S rRNA gene sequences were aligned with SINA Aligner [26], including 16S rRNA gene OTUs from Illumina sequencing, clone sequences, and archaea representative sequences obtained from ARB (Silva 138 database) [27]. Maximum-likelihood tree was inferred with RAxML (8.2.11) with rapid bootstrapping using the GTRGAMMA model [28]. The tree files were edited through iTOL software [29]. For phylogenomic tree, the concatenated set of 36 ribosomal protein genes based on the hidden Markov Model profile from Lee [30] were used for phylogenetic analyses in Anvi’o (6.1) [31]. Maximum-likelihood trees were built using IQ-TREE (1.6.12) [32] with the best-fit model and 1000 times ultrafast bootstrapping.

### Metagenomic assembly, genome binning, and gene annotation

Metagenomic analysis was followed by our previous study [17]. In brief, a total of ~1 µg DNA extracted from the original samples collected from Helgoland Mud area sediments with different depths (0–25 cm and 50–75 cm) and ~100 ng DNA from the DNA-SIP heavy fraction (density = 1.707 g/ml) were used for metagenomic sequencing on the Hiseq 4000 platform (2 × 150 bp) at Novogene (Cambridge, UK) [33]. The Ultra™ II DNA Library Prep Kit (New England Biolabs, USA) was used for metagenomic sequencing library preparation. The Metawrap package (1.2.1) [34] was employed to analyze the metagenomic reads. Briefly, quality-checked reads were trimmed and then assembled using Megahit (1.1.3) with the default settings [35]. Scaffolds (>1000 bps) were binned using a combination of MaxBin2 (2.2.6) [36], CONCOCT (1.0.0) [37], and metaBAT2 (2.12.1) [38]. The quality of the bins was improved by remapping the raw reads using short-read mapper BWA (0.7.17) [39] and reassembled using SPAdes (3.13.0) [40]. The completeness and contamination of MAGs were estimated by CheckM2 (0.1.3) [41]. Taxonomic classifications of archaeal MAGs were based on the GTDB database (0.3.3) [42]. Protein-coding regions were predicted using Prodigal (version 2.6.3) with the “-p meta” option [43]. The KEGG server (BlastKOALA) [44], eggNOG-mapper (5.0.0) [45], InterProScan tool (5.44-79.0) [46], and Diamond (0.9.22) vs. NCBI-nr database searched on April 2020 (E-value cutoff ≤1e-5) were used to annotate the protein-coding regions. The genes encoding proteins located outside of the cytoplasm were determined by using SignalP software (5.0b) with default parameters [47].

### Analysis of ^13^C-acetate, ethanol, and TOC

Concentrations and carbon isotope composition of acetate and ethanol were analyzed in the slurries’ supernatants of incubations amended with cellulose. Prior to analysis, 50 µl phosphoric acid were added to 200 µl of each supernatant retrieved from the incubations and incubated overnight to remove the high amount of ^13^C dissolved inorganic carbon. The δ^13^C values of acetate and ethanol were determined by liquid chromatography-isotope ratio mass spectrometry (LC-IRMS) according to approaches described previously by Heuer et al. [48]. The δ^13^C values of TOC were measured on a Flash 2000 elemental analyzer coupled with DELTA V Plus IRMS via a ConFlow II interface (EA-IRMS, Thermo Scientific, Bremen, Germany) [49]. Prior to analysis, dried sediment from 0.5-ml slurry was acidified using 1 ml HCl (37%) overnight to remove inorganic carbon and followed by evaporation for several days until HCl acid was fully evaporated.

### Analysis of methane and CO_2_

The concentration of methane and CO_2_ in the headspace of bottles was measured by gas chromatography as previously described [50]. The δ^13^C values of CO_2_ in the headspace were determined using a Thermo Finnigan Trace GC connected to a DELTA Plus XP IRMS (Thermo Scientific, Bremen, Germany) as described previously [51].

## Results and discussion

**Fig. 1:**
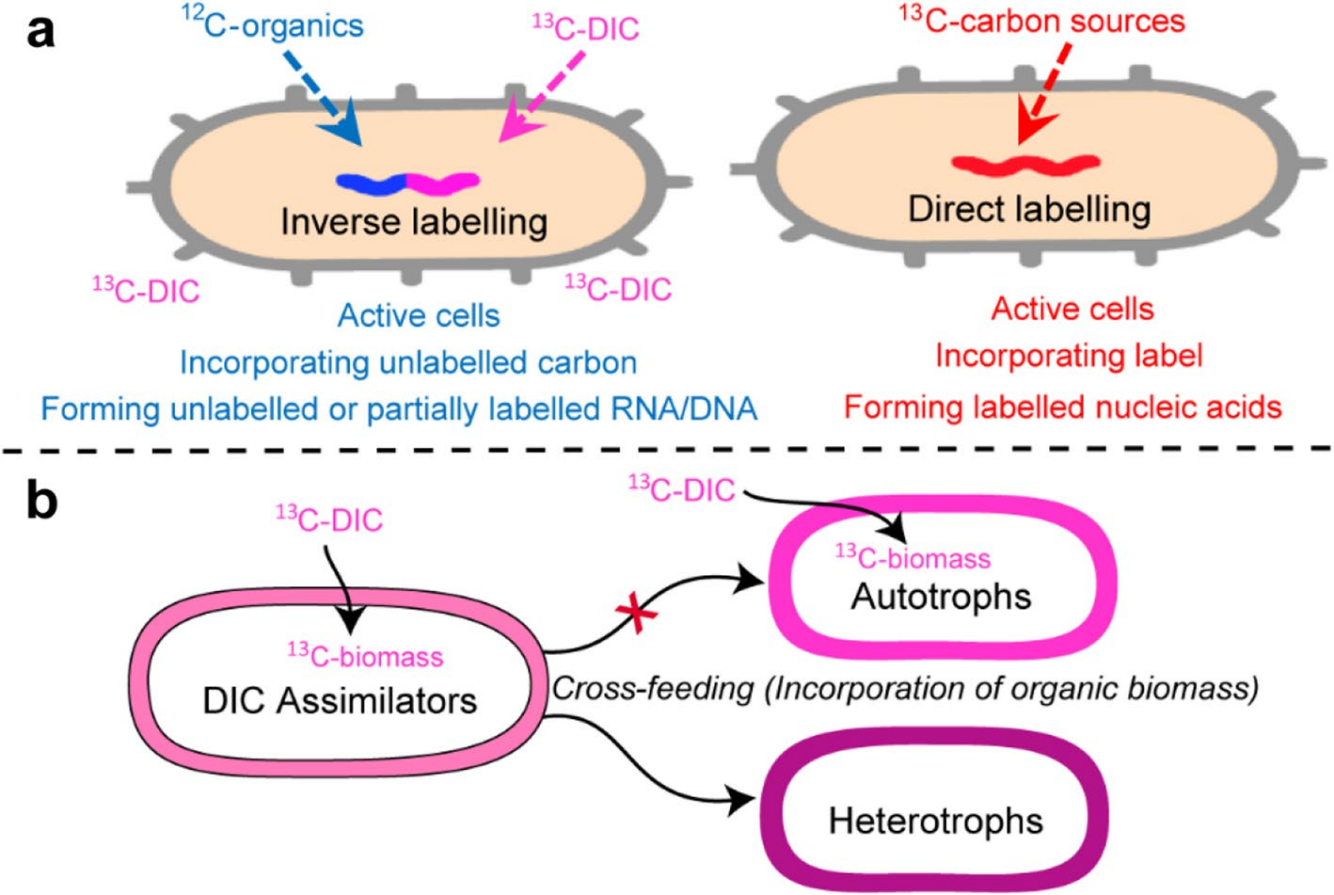
The strategy used in this study for identifying carbon utilization pattern during SIP. **a** Concept of inverse labeling for tracking unlabeled carbon assimilation compared to direct labeling. **b** Trophic strategies when cross-feeding occurs

We set up incubations of marine sediment close to *in situ* conditions with high concentrations of ^13^C-labeled DIC, reaching ~80% labeling level, to specifically track the incorporation of different organic compounds into rRNA and DNA of active archaea (“inverse labeling”; Fig. 1a), a similar concept adapted to the previous studies [52, 53]. This strategy aims to identify taxa that do not purely incorporate DIC but also use organic carbon substrates to build biomass (Fig. 1a). We focused on autotrophy while cross-feeding will not be a concern since autotrophs will not incorporate organic biomass (Fig. 1b). If cross-feeding is considered, the involved microbes have to be heterotrophs that assimilate produced biomass or metabolites although these organisms might have a low contribution (1–8% [54]) of inorganic carbon to the biomass via heterotrophic CO_2_ fixation (Fig. 1b).

### SIP revealed that unlabeled carbon was assimilated into nucleic acids by ANME-1 and Bathy-8

Triplicate SIP incubations were setup anaerobically by amending unlabeled organic substrates, ^13^C-DIC, and the potential electron acceptor lepidocrocite (an iron oxide γ-FeO(OH) commonly found in sediments [13]) due to the samples’ source from the methanic zone. Such setups can promote the stimulation of the fastidious ANME-1 and Bathy-8 archaea without enrichment, which can be detected by nucleic acid-SIP with its high sensitivity [55, 56]. As the amount of carbon from ^13^C-DIC in incubations was ~10 times higher than that of added unlabeled organics, the dilution effect of ^13^C-DIC by unlabeled DIC derived from organic carbon degradation is negligible (see methods), and thus, the signal of cross-feeding of CO_2_ from the cellulose oxidation will not be detected [15]. During incubation, δ^13^C-TOC (total organic carbon) development was tracked in order to define suitable sampling times for SIP (Fig. S2). Due to the slow growth rate of ANME-1 and the availability of ANME-1 MAGs from the original sediment, RNA-SIP was used for the ANME-1 experiment running for 255 days. In contrast, DNA-SIP in combination with metagenomics was performed for the Bathy-8 experiments because of the difficulty to retrieve MAGs from the original sediment due to their low abundance and high diversity. Here, we used the heavy fraction after incubating 386 days in the presence of lignin for obtaining MAGs (Table 1).

**Fig. 2:**
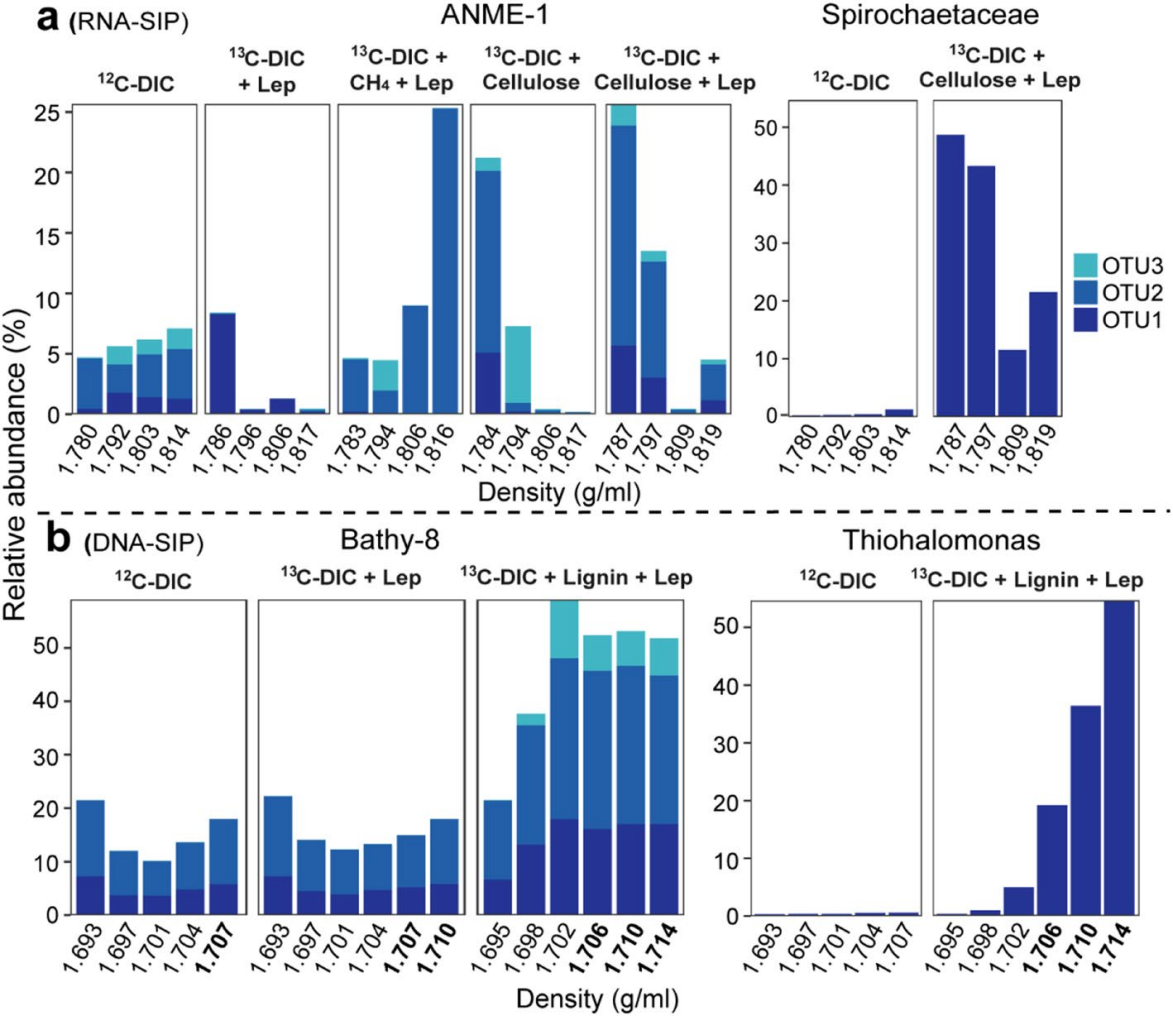
Identification of carbon assimilation patterns using inverse labeling of nucleic acid-SIP with methanic sediments from the Helgoland mud area. Relative abundance of 16S rRNA gene sequences of ANME-1 in total archaea and *Spirochaetaceae* in total bacteria from RNA-SIP gradient fractions (**a**), and Bathy-8 (percentage in archaea) and autotrophs *Thiohalomonas* (percentage in bacteria) from DNA-SIP gradient fractions (**b**). Densities from the heavy fractions of RNA- and DNA-SIP were marked in bold. DIC, dissolved inorganic carbon, i.e., bicarbonate; Lep, lepidocrocite. OTU1, OTU2, and OTU3 represent the most abundant operational taxonomic units for the individual microbe in b and c for each taxon (ANME-1, *Spirochaetaceae*, Bathy-8, and *Thiohalomonas*), respectively

RNA of ANME-1 was much more abundant in the isotopically “heavy” gradient fractions than in the “light” fractions from the incubations amended with ^13^C-DIC, CH_4_, and lepidocrocite (Fig. 2a, Fig. S3a), indicating labeling of RNA by ^13^C-DIC. Such carbon fixation by ANME-1 might owe to the sufficient input of electron acceptor, which was similar with the previous study showing that a high amount of inorganic carbon was assimilated into lipids in the presence of replete amounts of sulfate as electron acceptor [4]. However, ANME-1 was still active in incubations with cellulose in the absence of added methane. In those incubations, the relative abundance of RNA of ANME-1 in total archaea was substantially higher in the light fractions (~25%) than in the heavy fractions (below 5%) as well as in all fractions of the unlabeled DIC control (~5%); a similar pattern of labeling was observed for the distribution of heterotrophic microorganisms, i.e., cellulose degraders such as *Spirochaetaceae* [57] (Fig. 2a). In contrast, ANME-1 archaea were much less active in incubations amended with other organic polymers including lignin and humics (Fig. S4). Because RNA-SIP reflects the cell activity and thus RNA being newly synthesized, the unlabeled portion of carbon in RNA of ANME-1 archaea must have originated from the degradation of cellulose (Figs. 1a, 2a). Such unlabeled carbon assimilation was mainly derived from organic carbon instead of unlabeled DIC from cellulose for the following reasons: (i) ^13^C-DIC was amended at ten times higher concentration compared to the DIC that can potentially be liberated from unlabeled cellulose assuming full oxidation, and thus the DIC pool had a high ^13^C-labeling potential; (ii) amendment of cellulose (^13^C-DIC+Cellulose+Lep) strongly stimulated RNA accumulation of ANME-1 in light fractions compared to an incubation with methane (^13^C-DIC+CH_4_+Lep) (Fig. 2a), reflecting the utilization of unlabeled organic carbon.

For DNA-SIP samples in the Bathy-8 experiments, archaeal OTUs were indeed involved in degradation of lignin or possible intermediates and assimilation of inorganic carbon as their abundance was strongly increased in fractions containing ^13^C-DNA (density fractions, 1.698–1.714 g/ml) of incubations amended with lignin and ^13^C-DIC (Fig. 2b, Fig. S3a). This observation was consistent with a previous enrichment of Bathy-8 on lignin and a recent DNA-SIP study of carbon fixation by Bathy-8 [5, 58]. However, in contrast to the typical autotrophic *Thiohalomonas* (Fig. 2b, Fig. S5) [59, 60], the gradual decrease in relative abundance from the heavy to light fractions [61] was not observed for “autotrophic” Bathy-8 (Fig. 2b). Instead, Bathy-8 archaeal DNA abundance increased to the maximum in the density fraction of 1.702 g/ml (middle density fraction) while autotrophic *Thiohalomonas* had a very low abundance in the same fraction (Fig. 2b). The dilution of unlabeled background DNA of Bathy-8 archaea was ruled out as DNA of the newly identified OTU3 had a high abundance in the middle density fraction, and thus, this DNA was newly formed and partially labeled by incorporating unlabeled organic carbon and ^13^C-labeled DIC (Fig. 1a). Hence, the DNA of Bathy-8 shows a mixed carbon source signal, indicating these archaea incorporated both unlabeled lignin and ^13^C-labeled DIC into their biomass.

### Metagenomic analysis showed the link of catabolic and anabolic pathways for carbon utilization

**Fig. 3:**
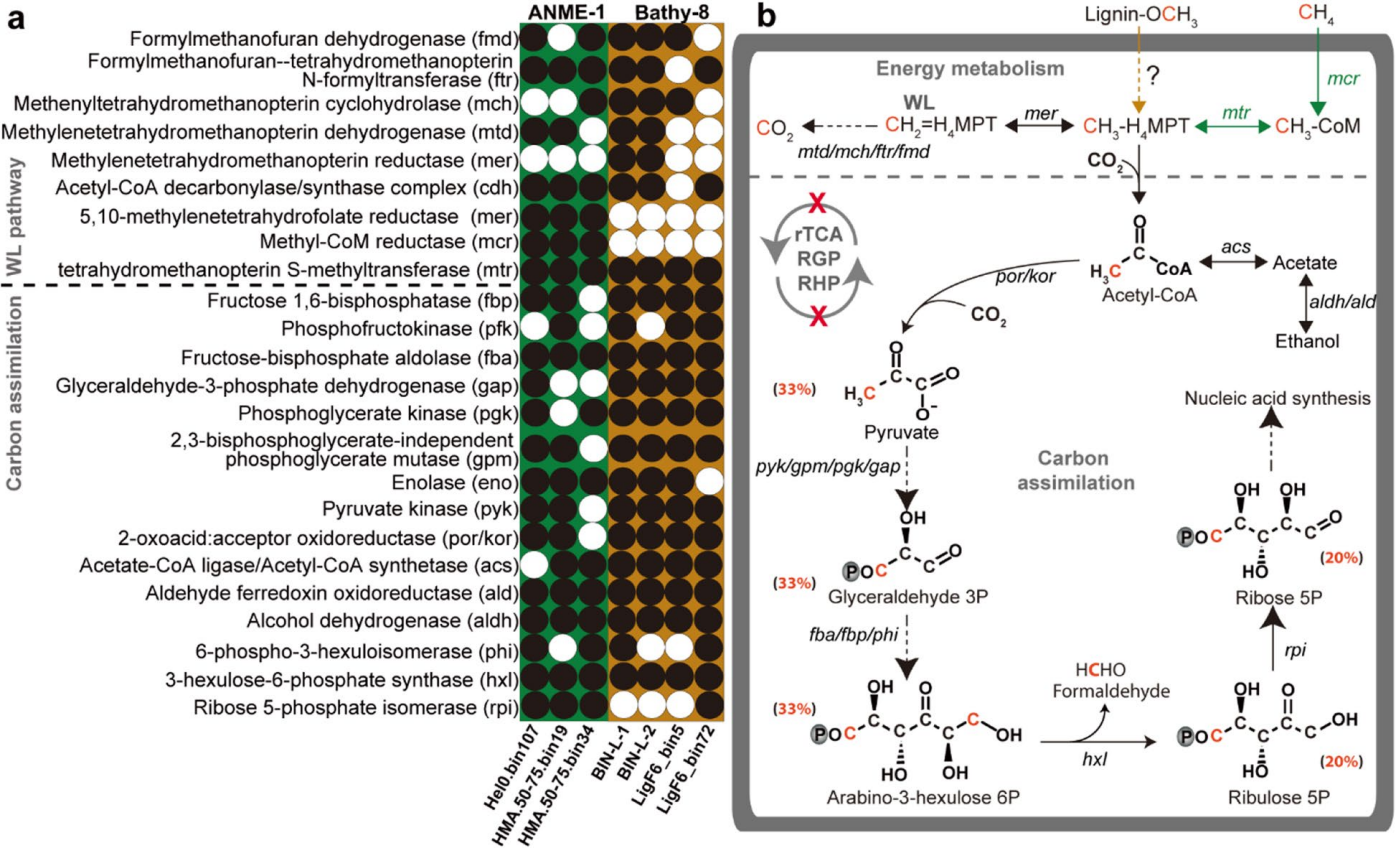
Pathway and carbon utilization pattern of ANME-1 and Bathy-8 archaea. Gene presence (**a**) and pathways involved in carbon utilization by ANME-1 and Bathy-8 with carbon contribution from organic carbon added besides the compounds (**b**). Black circle, presence of gene in MAG; white circle, absence of gene in MAG. Green arrows indicate the AOM pathway in ANME-1 and orange arrow indicates the putative transformation of methoxy groups by Bathy-8. Black arrows indicate the pathways for both ANME-1 and Bathy-8. Dashed arrows denote reactions catalyzed by multiple enzymes. The contribution of organic carbon to nucleic acids was based on the previous study of methylotrophic methanogens [56]. rTCA, reductive tricarboxylic acid cycle; RGP, reductive glycine pathway; RHP, reductive hexulose-phosphate pathway; WL, Wood–Ljungdahl pathway; CH_2_=H_4_MPT, methylene-tetrahydromethanopterin; CH_3_-H_4_MPT, methyl-tetrahydromethanopterin; CH_3_-CoM, methyl-coenzyme M

To support our SIP observations, we reconstructed the carbon utilization pathways based on our metagenomic results. We analyzed ANME-1 MAGs from the original sediment and Bathy-8 MAGs from the heavy DNA-SIP fraction (density fraction: 1.710 g/ml), as well as the previous enrichments with lignin for Bathy-8 [5] (Fig. S3, Table S1, see Methods). The multiple MAGs for each archaeal group were checked: 3 for ANME-1 and 4 for Bathy-8. We checked the phylogenetic identities of OTUs and clone sequences, together with the phylogenomic analysis of these MAGs, indicating their relevance to active OTUs (Fig. S3). The central energy and carbon metabolic pathways, including the Wood–Ljungdahl pathway (WL) and nucleic acid synthesis, were present in both archaeal types (Fig. 3). However, the other autotrophic pathways including reductive TCA cycle (rTCA), reductive glycine pathway (RGP) [62, 63], and reductive hexulose-phosphate pathway (RHP) [64] were highly incomplete in ANME-1 (Fig. 3b, Fig. S6). In particular, the features of carbon utilization in ANME-1 and Bathy-8 were obviously different from the typical synthetic chemoorganoautotrophs [8, 9], as we have indications of pathway interaction between energy metabolism and carbon assimilation in both archaea at the branching point of CH_3_-H_4_MPT, which can be used for both, energy and carbon metabolisms (Fig. 3). In contrast, the link between energy and carbon metabolism is disjointed in typical chemoorganoautotrophs [8, 9], and thus organic carbon acquisition for cell biomass synthesis is not possible during energy metabolism [8, 9]. Due to this metabolic link of organic carbon intermediates, organic carbon sources were utilized for biomass formation in a nonautotrophic fashion in ANME-1 and Bathy-8, and thus organic carbon assimilation was observed in the SIP incubations (Fig. 2a, b).

In our incubations without the typical amendment of sulfate, ANME-1 can utilize organic carbon, which is suggested by the following scenarios: (i) intermediates produced during cellulose degradation promote the growth of ANME-1. For example, given that acetate is an important carbon source for many obligate H_2_-dependent methanogens [65–67], and the genes encoding acetyl-CoA synthetase catalyzing reversible biosynthesis of acetyl-CoA from acetate were expressed [68], it is possible that ANME-1 assimilate intermediates such as acetate and ethanol as carbon source produced during cellulose degradation (Fig. 3, Table S2); It is also possible that ANME-1 utilize cell biomass for biosynthesis, a common activity has been identified in archaea [33, 69, 70]; (ii) as methanotrophs, ANME-1 archaea still can incorporate organic carbon from methane, as they feature the required pathways and low concentrations of methane (~0.8 µM in headspace) were available in incubations amended with cellulose (Fig. 3, Fig. S7). This is supported by the expression of the key gene (acetyl-CoA decarbonylase/synthase) encoding acetyl-CoA synthesis using methane (organic carbon) and inorganic carbon in a previous study [68].

For Bathy-8, it is still unclear how lignin is degraded. The activity of Bathy-8 archaea was only detected in the incubations amended with lignin but not protein, lipids, benzoate, fatty acids, and polysaccharide (laminarin) (Fig. S8) [59, 61, 71]. Degradation of aromatic compounds can be ruled out due to the lack of most genes encoding pathways for the degradation of lignin or its phenolic monomers (Fig. S6). Futher, utilization of these organic compounds would result in a heterotrophic or mixotrophic lifestyle instead of autotrophy [59, 61, 72]. Lignin is a common recalcitrant material in sediments giving its complex structure and low biodegradability. For example, lignin structure can be broken down by enzyme lignin peroxidases produced by fungi aerobically, which is very harsh in anaerobic condition. Our results rather suggest the possibility of utilizing methoxy groups, a typical functional group present in lignin, because ~1% of genes in the MAGs of Bathy-8 archaea encoded homologs of putative extracellular pectin lyase, which might be associated with the activity of demethylation (Table S3, S4) [73]. In line with our findings, more recent studies suggest that Bathy-8 archaea have potentials for the degradation of multiple aromatic methoxy compounds including 3,4-dimethoxybenzoate; vanillate; 2-methoxyphenol; 3,5-dimethoxybenzaldehyde; and vanillin [5, 74, 75]. However, it is still unclear if Bathy-8 archaea can directly utilize methoxy groups of lignin or have a syntrophic relation with other organisms for the utilization of the methoxy group of lignin monomers. Further work should specifically examine the role of Bathy-8 on lignin degradation based on enrichment cultivation and pure cultures.

For both, ANME-1 and Bathy-8, we searched for rTCA, RGP, and RHP genes. Regardless of MAG completeness, several genes encoding rTCA (pyruvate carboxylase, fumarate reductase, succinate-CoA-ligase for rTCA); RGP (formate-THF ligase, serine deaminase, glycine reductase); and RHP (phosphoribulokinase, D-arabino-3-hexulose-6-phospate synthase) are absent in all ANME-1 and Bathy-8 MAGs (Fig. S6), rendering carbon fixation unlikely by these autotrophic pathways. This is consistent with previous reports, which could not find these carbon fixation pathways [76, 77]. In addition, the reductive WL pathway for stepwise C1 reduction cannot be operative when acetyl-CoA, methane, or methoxyl group oxidation to CO_2_ is necessary for energy acquisition by running the WL in oxidative mode [78] (Fig. 3b).

For long-term SIP incubations, however, cross-feeding is a general concern. For example, the amendment of cellulose stimulated the growth of *Spirochaeta* 2 and FCPU426 bacteria while their biomass was unlabeled, indicating that the ^13^C-label was not significantly incorporated (Fig. 3b, Fig. S9). In the presence of lepidocrocite and ^13^C-DIC, ^13^C-labeled *Thiohalomonas* were detected (Fig. 2b, Fig. S5), showing a strong labeling level of amended ^13^C-DIC in SIP incubations and a clear-cut case of bacterial autotrophy. However, there is a potential for cross-feeding on bacterial biomass or metabolic products by ANME-1 and Bathy-8 no matter whether labeled or not; under these conditions, ANME-1 and Bathy-8 would be heterotrophs and not autotrophs (Fig. 1b). Another option would invoke isotopic equilibrium of ^13^C label from DIC, i.e., back-flux might have occurred in incubations with slow growth rates [79, 80], but this scenario cannot change the fact that the methyl group has been utilized as carbon source, which abiotically decrease the contribution of carbon from methyl group to biomass.

In our SIP incubations with very low energy yields for anaerobic oxidation of methane and lignin degradation, it seems difficult for microorganisms to fully gain energy for inorganic carbon assimilation and hence drive the utilization of intermediates from organic carbon degradation for biomass synthesis. Based on our metagenomic results, both ANME-1 and Bathy-8 show capacities of organic carbon assimilation, specifically C1 substrates derived from methane and methoxy groups in lignin, which has been also formerly suggested by natural isotope analysis and lipid SIP experiments [81–83]. This reflects a special nutritional category as C1 substrates can only provide the methyl group via CH_3_-H_4_MPT and an additional carbon from DIC is required for synthesizing carbon-carbon bonds. Nevertheless, both inorganic carbon and organic C1 compounds (methane or methanol) are important carbon sources for archaeal C1 utilizers (e.g., ANME-2a/c/d and methylotrophic methanogens) [80, 82, 84], while inorganic carbon is the main carbon source for nucleic acids when these C1 compounds and DIC are presented (Fig. 3b) [80].

## Conclusion

Our results evidence that ANME-1 and Bathy-8 archaea have the physiological versatility to utilize both organic and inorganic carbon sources. Thus, they are likely not sole chemoorganoautotrophs but rather more flexible in carbon assimilation. Given that there is a lack of mechanism about enigmatic chemoorganoautotrophs (ANME-1 and Bathy-8), our study developed the basic concept of microbial trophic strategy for understanding the uncultivated microorganisms in environments.

### Supplementary Information

**Supplementary Material 1.** 

## Data Availability

The archaeal MAGs data are available in the NCBI database under the project PRJNA505997 (Biosample SAMN14451653 and SAMN14451654) and PRJNA678468 (Biosample SAMN16802728 to SAMN16802739, SAMN20193292 and SAMN20193293). Sequencing data of SIP samples have been submitted to the Short Reads Archive under the project PRJNA505997.
