# Supplementary material for: Physiological versatility of ANME-1 and Bathyarchaeotoa-8 archaea evidenced by inverse stable isotope labeling
Source: Microbiome. 2024 Apr 3;12:68. doi: 10.1186/s40168-024-01779-z (PMC10988981; doi:10.1186/s40168-024-01779-z)
Supplement: Supplementary file 2 — Supplementary Material 1. [file 40168_2024_1779_MOESM1_ESM.pdf]

Physiological versatility of ANME-1 and Bathyarchaeotoa-8 archaea revealed by inverse stable isotope labelling

Xiuran Yin[1,2,3,4†*], Guowei Zhou[1,5], Mingwei Cai[6,7], Tim Richter-Heitmann[2], Qing-Zeng Zhu[4], Mara Maeke[2,3,4], Ajinkya C. Kulkarni[2], Rolf Nimzyk[2], Marcus Elvert[4,8], Michael W. Friedrich[2,4]

[1]State Key Laboratory of Marine Resource Utilization in South China Sea, Hainan University, Haikou, China

[2]Microbial Ecophysiology Group, Faculty of Biology/Chemistry, University of Bremen, Bremen, Germany.

[3]Max Planck Institute for Marine Microbiology, Bremen, Germany.

[4]MARUM - Center for Marine Environmental Sciences, University of Bremen, Bremen, Germany.

[5]School of Resources and Environmental Engineering, Anhui University, Hefei, Anhui, China.

[6]Institute of Chemical Biology, Shenzhen Bay Laboratory, Shenzhen, China.

[7]Archaeal Biology Center, Institute for Advanced Study, Shenzhen University, Shenzhen, China.

[8]Faculty of Geosciences, University of Bremen, Bremen, Germany.

[*]Correspondence:

Xiuran Yin, Faculty 02 (Chemistry/Biology) & MARUM, University of Bremen, Leobener Straße 3, D-28359, Bremen, Germany; E-mail: yin@uni-bremen.de; Tel: +49-421-218-63067.

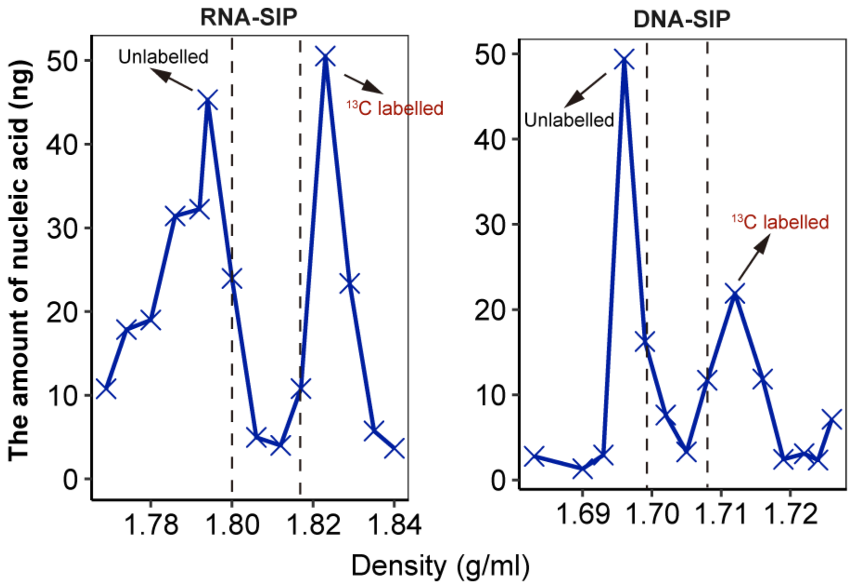

Fig. S1 RNA- and DNA-SIP profiles of the mixed equal amount of ^{12}C and ^{13}C labelled nucleic acids. The dashed lines indicate the density range for partially labelled fractions.

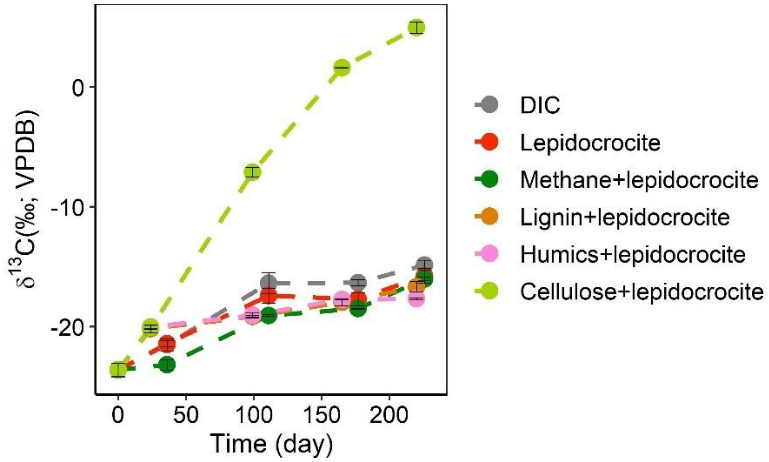

Fig. S2 The development of $\delta^{13}C$ values in total organic carbon. Since incorporation of ^{13}C-CO_2 reflects the microbial activity [1], we did not detect the microbial enrichment of Bathy-8 in the incubations amended with lignin and lepidocrocite. For ANME-1, the bacteria were enriched rather than ANME-1 (Fig.S9).

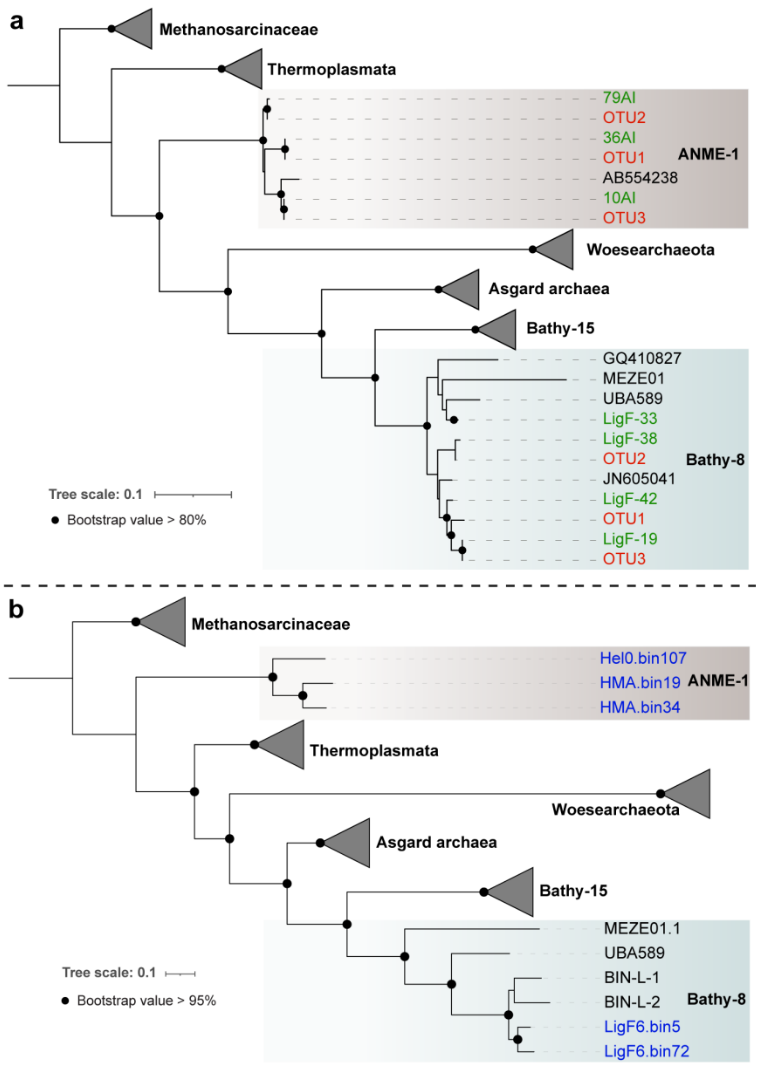

Fig. S3 Maximum likelihood tree of (a) archaeal 16S rRNA genes and (b) of 36 concatenated ribosomal proteins. OTUs, MAGs and clone sequences obtained in this study are marked in red, blue and green, respectively.

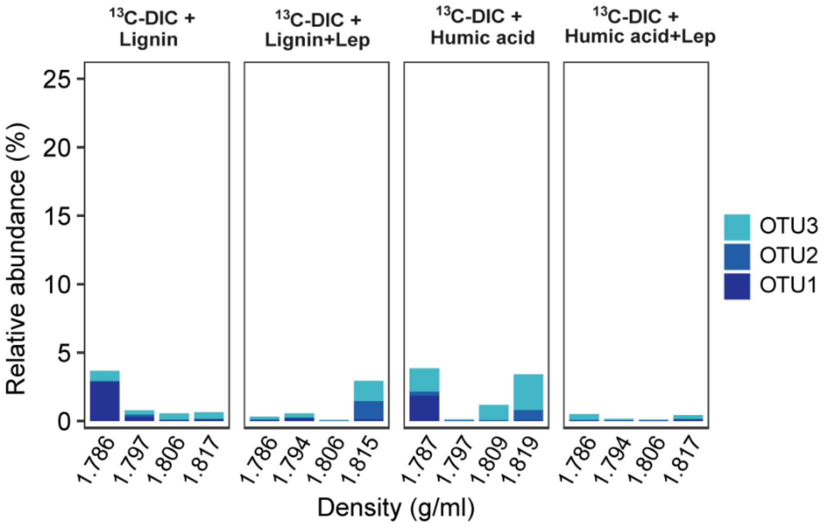

Fig. S4 Relative abundance of 16S rRNA gene sequences of ANME-1 in total archaea from DNA-SIP gradient fractions in incubations containing lignin or humic acids. Lep, lepidocrocite; DIC, dissolved inorganic carbon.

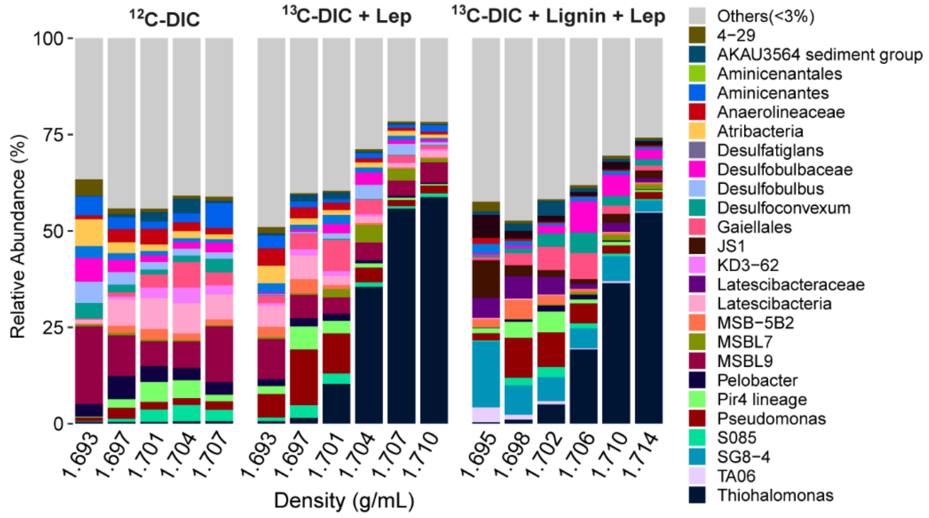

Fig. S5 Relative abundance of bacterial 16S rRNA gene sequences from DNA-SIP gradient fractions in incubations amended with ^{12}C-DIC, ^{13}C-DIC, lignin and lepidocrocite (Lep).

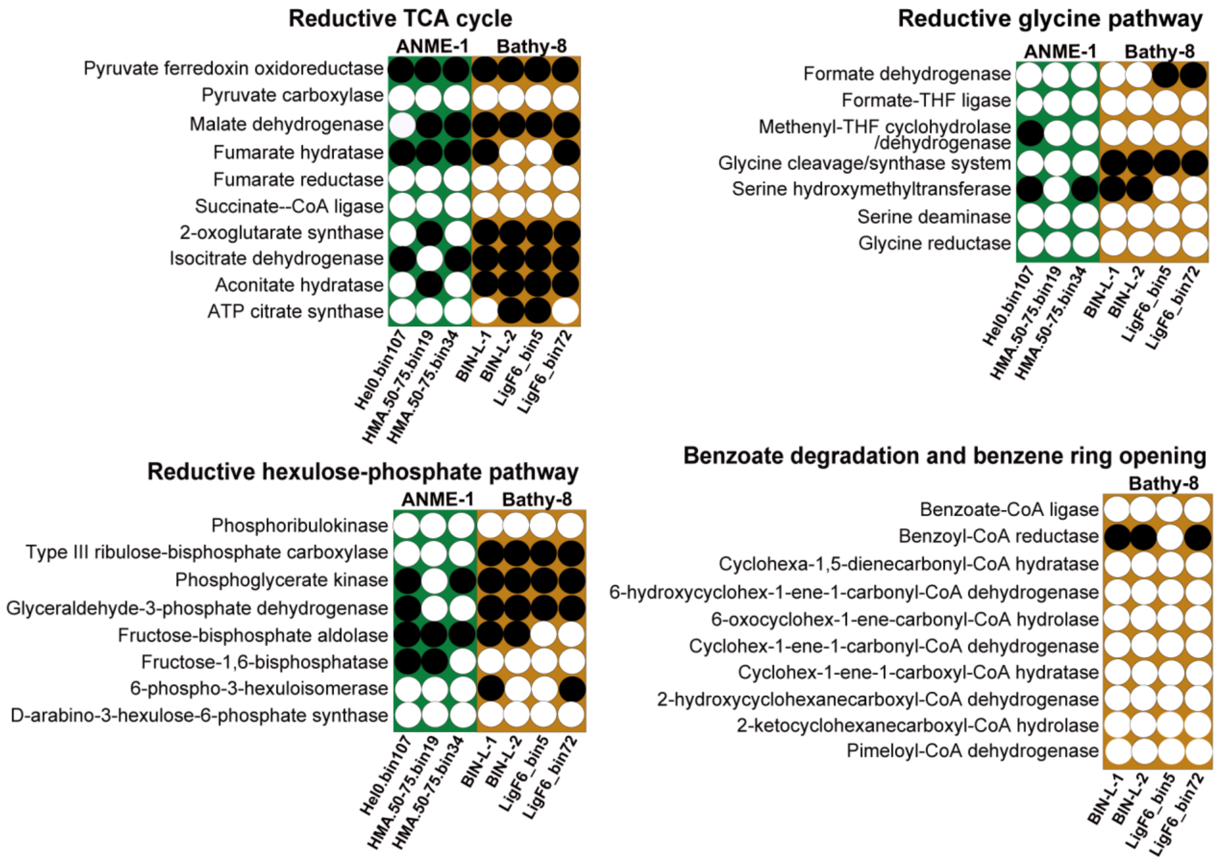

Fig. S6 Incomplete autotrophic pathways for ANME-1 and Bathy-8 archaea and benzoate degradation pathway for Bathy-8. Black circle: presence of gene in MAG; white circle: absence of gene in MAG.

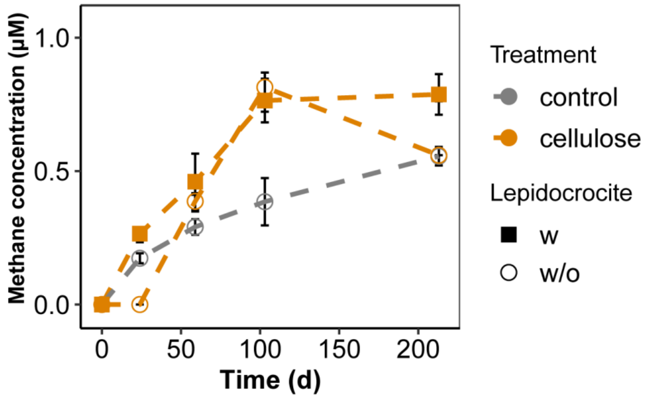

Fig. S7 Methane concentrations in the headspace of incubations amended with cellulose.

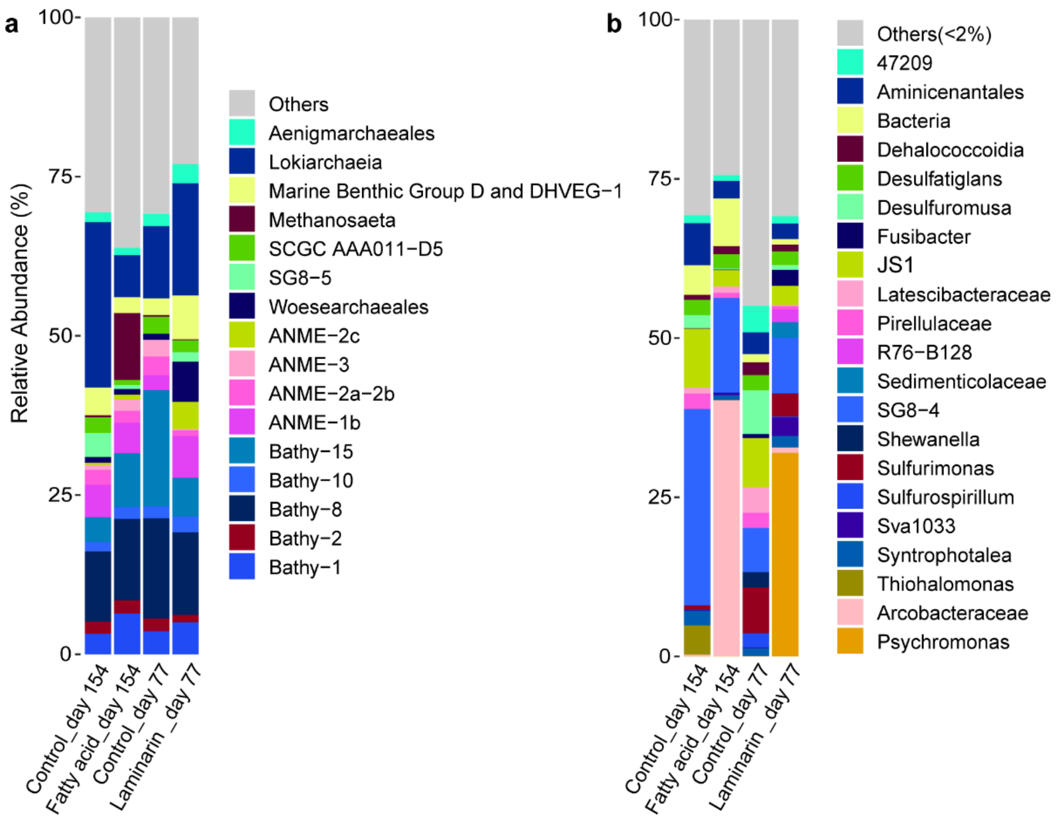

Fig. S8 Relative abundance of bacterial (a) and archaeal (b) 16S rRNA gene sequences from the slurry incubations amended with fatty acid (30 mM) and laminarin (~10 mg/l) using Helgoland Mud sediment. The results show that Bathy-8 and ANME-1 were not stimulated by fatty acid and laminarin in the long-term incubations.

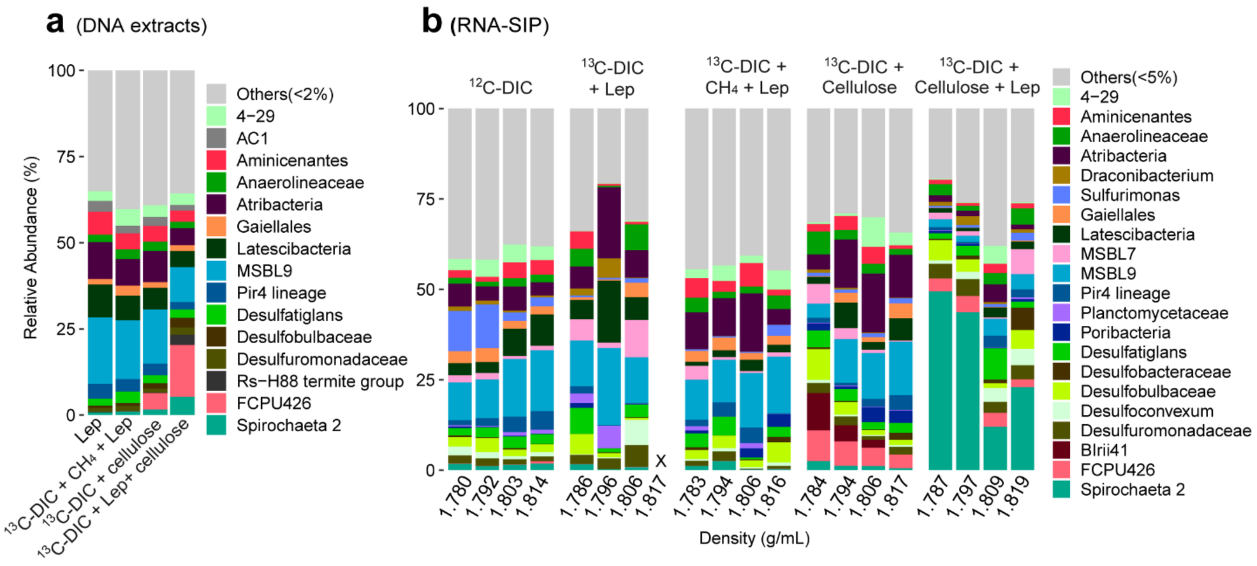

Fig. S9 Relative abundance of bacterial 16S rRNA gene sequences from DNA extracts (a, day 165) and RNA-SIP gradient fractions (b, day 225) in incubations amended with ^{12}C-DIC, ^{13}C-DIC, methane, cellulose and lepidocrocite (Lep). X indicates the low number of reads obtained from this SIP fraction during 16S rRNA gene sequencing.

Table S1 MAG information used in study.

MAG	Taxonomy	Completeness	Contamination	GC content	Origination
LigF.bin72	Bathy-8	51.63%	8.878%	43.1%	DNA-SIP heavy fraction from incubation amended with ^{13}C-bicarbonate, lignin and lepidocrocite using Helgoland mud sediment at depths from 238 to 263 cm
LigF.bin5	Bathy-8	54.98%	3.037%	43.3%	DNA-SIP heavy fraction from incubation amended with ^{13}C-bicarbonate, lignin and lepidocrocite using Helgoland mud sediment at depths from 238 to 263 cm
BIN-L-2*	Bathy-8	71.03%	1.32%	43.5%	Marine sediment incubation amended with lignin
BIN-L-1*	Bathy-8	82.47%	2.34%	41.65%	Marine sediment incubation amended with lignin
Hel0.bin107	ANME-1	77.94%	5.145%	41.9%	Helgoland mud sediment at depth from 16 to 41 cm
HMA50-75.bin19	ANME-1	61.36%	4.901%	46.8%	Helgoland mud sediment at depth from 50 to 75 cm (SMTZ)
HMA50-75.bin34	ANME-1	59.78%	0.73%	44.4%	Helgoland mud sediment at depth from 50 to 75 cm (SMTZ)

* indicates MAGs obtained from previous study [2].

Table S2 Concentration and **$\delta^{13}C$** of acetate or ethanol detected in incubations amended with cellulose.

Treatment	Component	Time (d)	Concentration (μM)	$\delta^{13}C$ (‰; VPDB)	^{13}C percentage (%)
^{13}C-DIC	acetate	165	NA	NA	NA
^{13}C-DIC	acetate	220	NA	NA	NA
^{13}C-DIC	ethanol	165	NA	NA	NA
^{13}C-DIC	ethanol	220	NA	NA	NA
Cellulose + ^{13}C-DIC	acetate	165	32.77 ± 2.66	3320 ± 80	4.61 ± 0.08
Cellulose + ^{13}C-DIC	acetate	220	51.94 ± 1.66	4230 ± 150	5.53 ± 0.15
Cellulose + ^{13}C-DIC + Lep	ethanol	165	21.28 ± 2.29	NA	NA
Cellulose + ^{13}C-DIC + Lep	ethanol	220	31.57 ± 3.16	490 ± 90	3.16 ± 0.10

Lep: lepidocrocite. This low level of ^{13}C in acetate and ethanol is not sufficient to promote density shift in RNA-SIP fractions [3].

Table S3 Number of genes potentially involved in methoxyl-group utilization by Bathy-8.

MAG	Number of homologous of pectin lyase fold/virulence factor like	Abundance of homologous of pectin lyase fold/virulence factor like (%)	Number of homologous of quinoprotein alcohol dehydrogenase-like	Abundance of homologous of quinoprotein alcohol dehydrogenase-like (%)
BIN-L-1	26	1.16	34	1.52
BIN-L-2	23	0.93	30	1.21
LigF6_bin5	9	0.67	11	0.81
LigF6_bin72	15	0.94	7	0.44

Table S4 Homologous of genes potentially involved in lignin degradation in Bathy-8 MAGs

Gene_ID	eggNOG.free.text.description	nr.annotation	interproscan_IPR	interproscan_annotation	Extracellular prediction
BIN-L-1_GCA_003096235.1_PIXT01000010.1_5	Periplasmic copper-binding protein (NosD)	hypothetical protein CW691 00280 [Candidatus Bathyarchaeota archaeon]	IPR022441	Parallel beta-helix repeat-2	SP(Sec/SPI)
BIN-L-1_GCA_003096235.1_PIXT01000124.1_4	NA	hypothetical protein CW691 06000 [Candidatus Bathyarchaeota archaeon]	IPR022441	Parallel beta-helix repeat-2	OTHER
BIN-L-1_GCA_003096235.1_PIXT01000161.1_3	COG3420 Nitrous oxidase accessory protein	hypothetical protein CW691 07570 [Candidatus Bathyarchaeota archaeon]	IPR022441	Parallel beta-helix repeat-2	SP(Sec/SPI)
BIN-L-1_GCA_003096235.1_PIXT01000163.1_2	NA	hypothetical protein CW691 07665 [Candidatus Bathyarchaeota archaeon]	IPR022441	Parallel beta-helix repeat-2	SP(Sec/SPI)
BIN-L-1_GCA_003096235.1_PIXT01000187.1_6	Belongs to the ompA family	hypothetical protein CW691 08945 [Candidatus Bathyarchaeota archaeon]	IPR022441	Parallel beta-helix repeat-2	SP(Sec/SPI)
BIN-L-1_GCA_003096235.1_PIXT01000252.1_3	NA	hypothetical protein CW691 11800 [Candidatus Bathyarchaeota archaeon]	IPR022441	Parallel beta-helix repeat-2	OTHER
BIN-L-2_GCA_003096255.1PIXS01000017.1_3	Domain present in carbohydrate binding proteins and sugar hydrolses	hypothetical protein CW716 00535 [Candidatus Bathyarchaeota archaeon]	IPR022441	Parallel beta-helix repeat-2	SP(Sec/SPI)
BIN-L-2_GCA_003096255.1PIXS01000049.1_2	Domain present in carbohydrate binding proteins and sugar hydrolses	hypothetical protein CW716 01470 [Candidatus Bathyarchaeota archaeon]	IPR022441	Parallel beta-helix repeat-2	SP(Sec/SPI)

BIN-L-2_GCA_003096255.1PIXS01000145.1_1	Belongs to the ompA family	hypothetical protein CW716 04445 [Candidatus Bathyarchaeota archaeon]	IPR022441	Parallel beta-helix repeat-2	SP(Sec/SPI)
BIN-L-2_GCA_003096255.1PIXS01000338.1_9	NA	hypothetical protein CW716 10480 [Candidatus Bathyarchaeota archaeon]	IPR022441	Parallel beta-helix repeat-2	SP(Sec/SPI)
LigF6_bin5_NODE_22083_length_4948_cov_2.406555_2	Periplasmic copper-binding protein (NosD)	hypothetical protein [Candidatus Bathyarchaeota archaeon]	IPR022441	Parallel beta-helix repeat-2	OTHER
LigF6_bin5_NODE_2313_length_17335_cov_2.110356_12	Periplasmic copper-binding protein (NosD)	hypothetical protein [Candidatus Bathyarchaeota archaeon]	IPR022441	Parallel beta-helix repeat-2	SP(Sec/SPI)
LigF6_bin5_NODE_28396_length_4267_cov_1.616908_3	Periplasmic copper-binding protein (NosD)	hypothetical protein [Candidatus Bathyarchaeota archaeon]	IPR022441	Parallel beta-helix repeat-2	OTHER
LigF6_bin72_NODE_19020_length_5392_cov_1.311491_2	Periplasmic copper-binding protein (NosD)	hypothetical protein CW716 00515 [Candidatus Bathyarchaeota archaeon]	IPR022441	Parallel beta-helix repeat-2	OTHER
LigF6_bin72_NODE_34879_length_3787_cov_0.666120_2	Periplasmic copper-binding protein (NosD)	hypothetical protein AC477 02605 [miscellaneous Crenarchaeota group-1 archaeon SG8-32-1]	IPR022441	Parallel beta-helix repeat-2	SP(Sec/SPI)
LigF6_bin72_NODE_5523_length_10813_cov_1.349803_2	Periplasmic copper-binding protein (NosD)	hypothetical protein D4R42 03845 [bacterium]	IPR022441	Parallel beta-helix repeat-2	OTHER
LigF6_bin72_NODE_6082_length_10288_cov_1.152347_13	NA	NA	IPR022441	Parallel beta-helix repeat-2	OTHER
BIN-L-1_GCA_003096235.1_PIXT01000011.1_2	Papain family cysteine protease	hypothetical protein CW691 00405 [Candidatus Bathyarchaeota archaeon]	IPR011050	Pectin lyase fold/virulence factor	SP(Sec/SPI)

BIN-L-1_GCA_003096235.1_PIXT01000014.1_3	NA	hypothetical protein CW691 00555 [Candidatus Bathyarchaeota archaeon]	IPR011050	Pectin lyase fold/virulence factor	SP(Sec/SPI)
BIN-L-1_GCA_003096235.1_PIXT01000035.1_2	NA	hypothetical protein CW691 01680 [Candidatus Bathyarchaeota archaeon]	IPR011050	Pectin lyase fold/virulence factor	SP(Sec/SPI)
BIN-L-1_GCA_003096235.1_PIXT01000035.1_3	Parallel beta-helix repeats	hypothetical protein CW691 01685 [Candidatus Bathyarchaeota archaeon]	IPR011050	Pectin lyase fold/virulence factor	SP(Sec/SPI)
BIN-L-1_GCA_003096235.1_PIXT01000035.1_4	NA	hypothetical protein CW691 01690, partial [Candidatus Bathyarchaeota archaeon]	IPR011050	Pectin lyase fold/virulence factor	OTHER
BIN-L-1_GCA_003096235.1_PIXT01000039.1_1	COG3420 Nitrous oxidase accessory protein	hypothetical protein CW691 01810, partial [Candidatus Bathyarchaeota archaeon]	IPR011050	Pectin lyase fold/virulence factor	SP(Sec/SPI)
BIN-L-1_GCA_003096235.1_PIXT01000089.1_14	NA	hypothetical protein CW691 04255 [Candidatus Bathyarchaeota archaeon]	IPR011050	Pectin lyase fold/virulence factor	SP(Sec/SPI)
BIN-L-1_GCA_003096235.1_PIXT01000102.1_14	Periplasmic copper-binding protein (NosD)	hypothetical protein CW691 05135 [Candidatus Bathyarchaeota archaeon]	IPR011050	Pectin lyase fold/virulence factor	SP(Sec/SPI)
BIN-L-1_GCA_003096235.1_PIXT01000158.1_6	Periplasmic copper-binding protein (NosD)	hypothetical protein CW691 07490 [Candidatus Bathyarchaeota archaeon]	IPR011050	Pectin lyase fold/virulence factor	OTHER
BIN-L-1_GCA_003096235.1_PIXT01000239.1_3	Right handed beta helix region	hypothetical protein CW691 11120 [Candidatus Bathyarchaeota archaeon]	IPR011050	Pectin lyase fold/virulence factor	SP(Sec/SPI)

BIN-L-1_GCA_003096235.1_PIXT01000252.1_1	Parallel beta-helix repeats	hypothetical protein CW691 11790 [Candidatus Bathyarchaeota archaeon]	IPR011050	Pectin lyase fold/virulence factor	SP(Sec/SPI)
BIN-L-1_GCA_003096235.1_PIXT01000253.1_4	Parallel beta-helix repeats	hypothetical protein CW691 11825 [Candidatus Bathyarchaeota archaeon]	IPR011050	Pectin lyase fold/virulence factor	OTHER
BIN-L-1_GCA_003096235.1_PIXT01000253.1_5	NA	hypothetical protein CW691 11830 [Candidatus Bathyarchaeota archaeon]	IPR011050	Pectin lyase fold/virulence factor	SP(Sec/SPI)
BIN-L-2_GCA_003096255.1PIXS01000016.1_3	Periplasmic copper-binding protein (NosD)	hypothetical protein CW716 00515 [Candidatus Bathyarchaeota archaeon]	IPR011050	Pectin lyase fold/virulence factor	SP(Sec/SPI)
BIN-L-2_GCA_003096255.1PIXS01000057.1_4	NA	hypothetical protein CW716 01775 [Candidatus Bathyarchaeota archaeon]	IPR011050	Pectin lyase fold/virulence factor	OTHER
BIN-L-2_GCA_003096255.1PIXS01000130.1_1	Periplasmic copper-binding protein (NosD)	hypothetical protein CW716 03860, partial [Candidatus Bathyarchaeota archaeon]	IPR011050	Pectin lyase fold/virulence factor	SP(Sec/SPI)
BIN-L-2_GCA_003096255.1PIXS01000131.1_2	Parallel beta-helix repeats	hypothetical protein CW716 03885 [Candidatus Bathyarchaeota archaeon]	IPR011050	Pectin lyase fold/virulence factor	SP(Sec/SPI)
BIN-L-2_GCA_003096255.1PIXS01000154.1_3	NA	hypothetical protein CW716 04815 [Candidatus Bathyarchaeota archaeon]	IPR011050	Pectin lyase fold/virulence factor	SP(Sec/SPI)
BIN-L-2_GCA_003096255.1PIXS01000154.1_4	NA	hypothetical protein CW716 04820 [Candidatus Bathyarchaeota archaeon]	IPR011050	Pectin lyase fold/virulence factor	SP(Sec/SPI)

BIN-L-2_GCA_003096255.1PIXS01000171.1_1	Right handed beta helix region	hypothetical protein CW716 05285, partial [Candidatus Bathyarchaeota archaeon]	IPR011050	Pectin lyase fold/virulence factor	OTHER
BIN-L-2_GCA_003096255.1PIXS01000182.1_1	Parallel beta-helix repeats	hypothetical protein CW716 05705, partial [Candidatus Bathyarchaeota archaeon]	IPR011050	Pectin lyase fold/virulence factor	SP(Sec/SPI)
BIN-L-2_GCA_003096255.1PIXS01000187.1_3	NA	hypothetical protein CW716 05835 [Candidatus Bathyarchaeota archaeon]	IPR011050	Pectin lyase fold/virulence factor	SP(Sec/SPI)
BIN-L-2_GCA_003096255.1PIXS01000207.1_1	NA	hypothetical protein CW691 00405 [Candidatus Bathyarchaeota archaeon]	IPR011050	Pectin lyase fold/virulence factor	SP(Sec/SPI)
BIN-L-2_GCA_003096255.1PIXS01000268.1_4	NA	hypothetical protein CW716 08325 [Candidatus Bathyarchaeota archaeon]	IPR011050	Pectin lyase fold/virulence factor	SP(Sec/SPI)
BIN-L-2_GCA_003096255.1PIXS01000301.1_1	NA	hypothetical protein CW716 09285 [Candidatus Bathyarchaeota archaeon]	IPR011050	Pectin lyase fold/virulence factor	SP(Sec/SPI)
BIN-L-2_GCA_003096255.1PIXS01000338.1_10	NA	hypothetical protein CW716 10485 [Candidatus Bathyarchaeota archaeon]	IPR011050	Pectin lyase fold/virulence factor	OTHER
BIN-L-2_GCA_003096255.1PIXS01000377.1_1	NA	hypothetical protein CW716 11630, partial [Candidatus Bathyarchaeota archaeon]	IPR011050	Pectin lyase fold/virulence factor	OTHER
BIN-L-2_GCA_003096255.1PIXS01000409.1_6	Parallel beta-helix repeats	hypothetical protein CW716 12625, partial [Candidatus Bathyarchaeota archaeon]	IPR011050	Pectin lyase fold/virulence factor	OTHER

BIN-L-2_GCA_003096255.1PIXS01000419.1_5	NA	hypothetical protein CW71612935 [Candidatus Bathyarchaeota archaeon]	IPR011050	Pectin lyase fold/virulence factor	SP(Sec/SPI)
LigF6_bin5_NODE_11268_length_7260_cov_2.444273_5	Periplasmic copper-binding protein (NosD)	hypothetical protein CW71604345 [Candidatus Bathyarchaeota archaeon]	IPR011050	Pectin lyase fold/virulence factor	SP(Sec/SPI)
LigF6_bin5_NODE_23214_length_4803_cov_2.116766_1	NA	hypothetical protein [Candidatus Bathyarchaeota archaeon]	IPR011050	Pectin lyase fold/virulence factor	SP(Sec/SPI)
LigF6_bin5_NODE_32783_length_3928_cov_2.993949_3	NA	hypothetical protein D4R4202295 [bacterium]	IPR011050	Pectin lyase fold/virulence factor	SP(Sec/SPI)
LigF6_bin5_NODE_4192_length_12620_cov_2.217642_4	Periplasmic copper-binding protein (NosD)	hypothetical protein AC47702605 [miscellaneous Crenarchaeota group-1 archaeon SG8-32-1]	IPR011050	Pectin lyase fold/virulence factor	SP(Sec/SPI)
LigF6_bin5_NODE_44043_length_3308_cov_2.624332_1	NA	right-handed parallel beta-helix repeat-containing protein [Candidatus Bathyarchaeota archaeon]	IPR011050	Pectin lyase fold/virulence factor	OTHER
LigF6_bin72_NODE_14810_length_6237_cov_1.492635_6	NA	hypothetical protein CW69111790 [Candidatus Bathyarchaeota archaeon]	IPR011050	Pectin lyase fold/virulence factor	OTHER
LigF6_bin72_NODE_27374_length_4361_cov_1.382853_2	NA	hypothetical protein DRO6914075 [Candidatus Bathyarchaeota archaeon]	IPR011050	Pectin lyase fold/virulence factor	SP(Sec/SPI)
LigF6_bin72_NODE_31616_length_4011_cov_0.899331_1	NA	hypothetical protein E3J7306970, partial [Candidatus Bathyarchaeota archaeon]	IPR011050	Pectin lyase fold/virulence factor	SP(Sec/SPI)

LigF6_bin72_NODE_3204_length_14669_cov_1.387980_11	NA	hypothetical protein E3J73 00420 [Candidatus Bathyarchaeota archaeon]	IPR011050	Pectin lyase fold/virulence factor	SP(Sec/SPI)
LigF6_bin72_NODE_32950_length_3918_cov_1.659720_2	NA	hypothetical protein CW716 11635 [Candidatus Bathyarchaeota archaeon]	IPR011050	Pectin lyase fold/virulence factor	SP(Sec/SPI)
LigF6_bin72_NODE_34879_length_3787_cov_0.666120_1	Periplasmic copper-binding protein (NosD)	hypothetical protein [Candidatus Bathyarchaeota archaeon]	IPR011050	Pectin lyase fold/virulence factor	SP(Sec/SPI)
LigF6_bin72_NODE_43006_length_3356_cov_0.614432_4	Nitrous oxidase accessory protein	hypothetical protein [Candidatus Bathyarchaeota archaeon]	IPR011050	Pectin lyase fold/virulence factor	OTHER
LigF6_bin72_NODE_4874_length_11593_cov_1.552241_8	Right handed beta helix region	hypothetical protein E3J73 08030 [Candidatus Bathyarchaeota archaeon]	IPR011050	Pectin lyase fold/virulence factor	SP(Sec/SPI)
LigF6_bin72_NODE_53460_length_2963_cov_1.104725_1	NA	hypothetical protein CW691 05135 [Candidatus Bathyarchaeota archaeon]	IPR011050	Pectin lyase fold/virulence factor	OTHER
LigF6_bin72_NODE_5523_length_10813_cov_1.349803_6	Right handed beta helix region	hypothetical protein CW691 00280 [Candidatus Bathyarchaeota archaeon]	IPR011050	Pectin lyase fold/virulence factor	#
BIN-L-1_GCA_003096235.1_PIXT01000047.1_6	Papain family cysteine protease	hypothetical protein CW691 02095 [Candidatus Bathyarchaeota archaeon]	IPR007742	Periplasmic copper-binding protein NosD, beta helix domain	SP(Sec/SPI)
BIN-L-1_GCA_003096235.1_PIXT01000047.1_7	NA	hypothetical protein CW691 02100 [Candidatus Bathyarchaeota archaeon]	IPR007742	Periplasmic copper-binding protein NosD, beta helix domain	SP(Sec/SPI)
BIN-L-1_GCA_003096235.1_PIXT01000090.1_6	NA	hypothetical protein CW691 04290	IPR007742	Periplasmic copper-binding	SP(Sec/SPI)

		[Candidatus Bathyarchaeota archaeon]		protein NosD, beta helix domain	
BIN-L-1_GCA_003096235.1_PIXT01000111.1_2	NA	hypothetical protein CW691 05530 [Candidatus Bathyarchaeota archaeon]	IPR007742	Periplasmic copper-binding protein NosD, beta helix domain	OTHER
BIN-L-1_GCA_003096235.1_PIXT01000125.1_3	NA	hypothetical protein CW691 06015 [Candidatus Bathyarchaeota archaeon]	IPR007742	Periplasmic copper-binding protein NosD, beta helix domain	SP(Sec/SPI)
BIN-L-1_GCA_003096235.1_PIXT01000144.1_7	Periplasmic copper-binding protein (NosD)	hypothetical protein CW691 06830 [Candidatus Bathyarchaeota archaeon]	IPR007742	Periplasmic copper-binding protein NosD, beta helix domain	SP(Sec/SPI)
BIN-L-1_GCA_003096235.1_PIXT01000157.1_22	Domain present in carbohydrate binding proteins and sugar hydrolses	hypothetical protein CW691 07370 [Candidatus Bathyarchaeota archaeon]	IPR007742	Periplasmic copper-binding protein NosD, beta helix domain	SP(Sec/SPI)
BIN-L-1_GCA_003096235.1_PIXT01000179.1_7	NA	hypothetical protein CW691 08615 [Candidatus Bathyarchaeota archaeon]	IPR007742	Periplasmic copper-binding protein NosD, beta helix domain	SP(Sec/SPI)
BIN-L-1_GCA_003096235.1_PIXT01000207.1_8	Periplasmic copper-binding protein (NosD)	hypothetical protein CW691 10035 [Candidatus Bathyarchaeota archaeon]	IPR007742	Periplasmic copper-binding protein NosD, beta helix domain	SP(Sec/SPI)
BIN-L-1_GCA_003096235.1_PIXT01000253.1_7	NA	hypothetical protein CW691 11840 [Candidatus Bathyarchaeota archaeon]	IPR007742	Periplasmic copper-binding protein NosD, beta helix domain	SP(Sec/SPI)
BIN-L-2_GCA_003096255.1PIXS01000049.1_3	pectinesterase activity	hypothetical protein CW716 01475 [Candidatus Bathyarchaeota archaeon]	IPR007742	Periplasmic copper-binding protein NosD, beta helix domain	OTHER
BIN-L-2_GCA_003096255.1PIXS01000068.1_1	COG4677 Pectin methylesterase	hypothetical protein CW716 02050, partial [Candidatus	IPR007742	Periplasmic copper-binding protein	OTHER

		Bathyarchaeota archaeon]		NosD, beta helix domain	
BIN-L-2_GCA_003096255.1PIXS01000076.1_5	Periplasmic copper-binding protein (NosD)	hypothetical protein CW716 02240 [Candidatus Bathyarchaeota archaeon]	IPR007742	Periplasmic copper-binding protein NosD, beta helix domain	OTHER
BIN-L-2_GCA_003096255.1PIXS01000082.1_4	amino acid activation for nonribosomal peptide biosynthetic process	hypothetical protein CW716 02410 [Candidatus Bathyarchaeota archaeon]	IPR007742	Periplasmic copper-binding protein NosD, beta helix domain	OTHER
BIN-L-2_GCA_003096255.1PIXS01000085.1_9	NA	hypothetical protein CW716 02495, partial [Candidatus Bathyarchaeota archaeon]	IPR007742	Periplasmic copper-binding protein NosD, beta helix domain	OTHER
BIN-L-2_GCA_003096255.1PIXS01000143.1_1	NA	hypothetical protein CW716 04345 [Candidatus Bathyarchaeota archaeon]	IPR007742	Periplasmic copper-binding protein NosD, beta helix domain	OTHER
BIN-L-2_GCA_003096255.1PIXS01000172.1_8	Papain family cysteine protease	hypothetical protein CW716 05405 [Candidatus Bathyarchaeota archaeon]	IPR007742	Periplasmic copper-binding protein NosD, beta helix domain	SP(Sec/SPI)
BIN-L-2_GCA_003096255.1PIXS01000177.1_1	Right handed beta helix region	hypothetical protein CW716 05525, partial [Candidatus Bathyarchaeota archaeon]	IPR007742	Periplasmic copper-binding protein NosD, beta helix domain	OTHER
BIN-L-2_GCA_003096255.1PIXS01000250.1_6	Periplasmic copper-binding protein (NosD)	hypothetical protein CW716 07665 [Candidatus Bathyarchaeota archaeon]	IPR007742	Periplasmic copper-binding protein NosD, beta helix domain	SP(Sec/SPI)
BIN-L-2_GCA_003096255.1PIXS01000268.1_3	NA	hypothetical protein CW716 08320 [Candidatus Bathyarchaeota archaeon]	IPR007742	Periplasmic copper-binding protein NosD, beta helix domain	SP(Sec/SPI)
BIN-L-2_GCA_003096255.1PIXS01000268.1_5	Periplasmic copper-binding protein (NosD)	hypothetical protein CW716 08330 [Candidatus	IPR007742	Periplasmic copper-binding protein	SP(Sec/SPI)

		Bathyarchaeota archaeon]		NosD, beta helix domain	
BIN-L-2_GCA_003096255.1PIXS01000289.1_4	Carbohydrate-binding and sugar hydrolysis	hypothetical protein CW716 08990, partial [Candidatus Bathyarchaeota archaeon]	IPR007742	Periplasmic copper-binding protein NosD, beta helix domain	OTHER
BIN-L-2_GCA_003096255.1PIXS01000353.1_2	Carbohydrate-binding and sugar hydrolysis	hypothetical protein CW716 10930 [Candidatus Bathyarchaeota archaeon]	IPR007742	Periplasmic copper-binding protein NosD, beta helix domain	SP(Sec/SPI)
LigF6_bin5_NODE_26784_length_4416_cov_2.261599_2	Periplasmic copper-binding protein (NosD)	hypothetical protein [Candidatus Bathyarchaeota archaeon]	IPR007742	Periplasmic copper-binding protein NosD, beta helix domain	SP(Sec/SPI)
LigF6_bin5_NODE_35073_length_3774_cov_2.082534_1	NA	DUF1565 domain-containing protein [Candidatus Bathyarchaeota archaeon]	IPR007742	Periplasmic copper-binding protein NosD, beta helix domain	OTHER
LigF6_bin5_NODE_4960_length_11478_cov_1.781869_2	NA	hypothetical protein [Candidatus Bathyarchaeota archaeon]	IPR007742	Periplasmic copper-binding protein NosD, beta helix domain	SP(Sec/SPI)
LigF6_bin5_NODE_6928_length_9547_cov_1.736306_10	NA	hypothetical protein AC477 02605 [miscellaneous Crenarchaeota group-1 archaeon SG8-32-1]	IPR007742	Periplasmic copper-binding protein NosD, beta helix domain	OTHER
LigF6_bin72_NODE_114451_length_1942_cov_0.750413_1	NA	hypothetical protein E3J73 08030 [Candidatus Bathyarchaeota archaeon]	IPR007742	Periplasmic copper-binding protein NosD, beta helix domain	SP(Sec/SPI)
LigF6_bin72_NODE_13393_length_6598_cov_0.916705_2	NA	hypothetical protein [Candidatus Bathyarchaeota archaeon]	IPR007742	Periplasmic copper-binding protein NosD, beta helix domain	SP(Sec/SPI)
LigF6_bin72_NODE_28995_length_4216_cov_1.571533_5	NA	hypothetical protein	IPR007742	Periplasmic copper-	SP(Sec/SPI)

		[Candidatus Bathyarchaeota archaeon]		binding protein NosD, beta helix domain	
LigF6_bin72_NODE_31616_length_4011_cov_0.899331_2	Periplasmic copper-binding protein (NosD)	hypothetical protein D4R42 02305 [bacterium]	IPR007742	Periplasmic copper-binding protein NosD, beta helix domain	SP(Sec/SPI)
LigF6_bin72_NODE_48159_length_3143_cov_1.077255_1	NA	hypothetical protein CW716 01470 [Candidatus Bathyarchaeota archaeon]	IPR007742	Periplasmic copper-binding protein NosD, beta helix domain	OTHER
LigF6_bin72_NODE_53306_length_2968_cov_0.844773_3	NA	hypothetical protein CW716 11630, partial [Candidatus Bathyarchaeota archaeon]	IPR007742	Periplasmic copper-binding protein NosD, beta helix domain	OTHER
LigF6_bin72_NODE_5876_length_10481_cov_1.330114_1	NA	hypothetical protein CW691 00555 [Candidatus Bathyarchaeota archaeon]	IPR007742	Periplasmic copper-binding protein NosD, beta helix domain	OTHER
LigF6_bin72_NODE_6082_length_10288_cov_1.152347_12	Periplasmic copper-binding protein (NosD)	hypothetical protein E3J73 06970, partial [Candidatus Bathyarchaeota archaeon]	IPR007742	Periplasmic copper-binding protein NosD, beta helix domain	SP(Sec/SPI)
LigF6_bin72_NODE_7874_length_8878_cov_1.078391_1	NA	hypothetical protein D4R42 02295 [bacterium]	IPR007742	Periplasmic copper-binding protein NosD, beta helix domain	SP(Sec/SPI)
LigF6_bin72_NODE_81396_length_2340_cov_1.413918_3	Right handed beta helix region	hypothetical protein [Candidatus Bathyarchaeota archaeon]	IPR007742	Periplasmic copper-binding protein NosD, beta helix domain	OTHER
BIN-L-1_GCA_003096235.1_PIXT01000102.1_12	Periplasmic copper-binding protein (NosD)	hypothetical protein CW691 05125 [Candidatus Bathyarchaeota archaeon]	IPR039448	Right handed beta helix domain	OTHER
BIN-L-1_GCA_003096235.1_PIXT01000205.1_2	Parallel beta-helix repeats	hypothetical protein CW691 09950	IPR039448	Right handed beta helix domain	SP(Sec/SPI)

		[Candidatus Bathyarchaeota archaeon]			
BIN-L-1_GCA_003096235.1_PIXT01000252.1_2	Periplasmic copper-binding protein (NosD)	hypothetical protein CW691 11795 [Candidatus Bathyarchaeota archaeon]	IPR039448	Right handed beta helix domain	SP(Sec/SPI)
BIN-L-1_GCA_003096235.1_PIXT01000253.1_6	NA	hypothetical protein CW691 11835 [Candidatus Bathyarchaeota archaeon]	IPR039448	Right handed beta helix domain	SP(Sec/SPI)
BIN-L-2_GCA_003096255.1PIXS01000154.1_5	NA	hypothetical protein CW716 04825, partial [Candidatus Bathyarchaeota archaeon]	IPR039448	Right handed beta helix domain	SP(Sec/SPI)
BIN-L-2_GCA_003096255.1PIXS01000225.1_1	Bacterial Ig-like domain (group 3)	hypothetical protein CW716 06945 [Candidatus Bathyarchaeota archaeon]	IPR039448	Right handed beta helix domain	SP(Sec/SPI)
BIN-L-2_GCA_003096255.1PIXS01000377.1_2	Periplasmic copper-binding protein (NosD)	hypothetical protein CW716 11635 [Candidatus Bathyarchaeota archaeon]	IPR039448	Right handed beta helix domain	SP(Sec/SPI)
LigF6_bin5_NODE_36552_length_3686_cov_2.389154_2	Periplasmic copper-binding protein (NosD)	hypothetical protein CW716 12935 [Candidatus Bathyarchaeota archaeon]	IPR039448	Right handed beta helix domain	SP(Sec/SPI)
LigF6_bin5_NODE_6867_length_9593_cov_3.038559_6	NA	hypothetical protein [Candidatus Bathyarchaeota archaeon]	IPR039448	Right handed beta helix domain	SP(Sec/SPI)
LigF6_bin72_NODE_25190_length_4576_cov_1.026748_2	NA	hypothetical protein [Candidatus Bathyarchaeota archaeon]	IPR039448	Right handed beta helix domain	OTHER
LigF6_bin72_NODE_79847_length_2365_cov_0.996425_4	NA	hypothetical protein CW691 07570 [Candidatus Bathyarchaeota archaeon]	IPR039448	Right handed beta helix domain	SP(Sec/SPI)

NA: not available.

Reference

1. Roslev P, Larsen MB, Jorgensen D, Hesselsoe M. Use of heterotrophic CO_2 assimilation as a measure of metabolic activity in planktonic and sessile bacteria. J Microbiol Methods. 2004;59:381-93.
2. Yu T, Wu W, Liang W, Lever MA, Hinrichs KU, Wang F. Growth of sedimentary Bathyarchaeota on lignin as an energy source. Proc Natl Acad Sci USA. 2018;115:6022-7.
3. Manefield M, Whiteley AS, Ostle N, Ineson P, Bailey MJ. Technical considerations for RNA-based stable isotope probing an approach to associating microbial diversity with microbial community function. Rapid Commun Mass Spectrom. 2002;16:2179-83.
